# Interlaboratory comparison of amyloid‐beta [AB1‐42], [AB1‐40] and its ratio derived from CSF at two reference laboratories using the lumipulse platform

**DOI:** 10.1002/alz70856_103706

**Published:** 2025-12-24

**Authors:** James Arthur Rock, Anna McGuffin, Tao Han, Shaofeng Zhang, Fred Kim, Rachel R. Radwan, Francesca De Simone, Natalya Benina, Jai Jun Choung, Saeed A Jortani

**Affiliations:** ^1^ AriBio Co., Ltd, San Diego, CA, USA; ^2^ KCTL, Louisville, KY, USA; ^3^ Teddy Clinical Research Laboratory, Yangpu District, Shanghai, China; ^4^ AriBio Co., Ltd., San Diego, CA, USA; ^5^ Fujirebio Diagnostics Inc, Malvern, PA, USA; ^6^ AriBio Co., Ltd., Seongnam‐si, Gyeonggi‐do, Korea, Republic of (South); ^7^ University of Louisville School of Medicine, Louisville, KY, USA

## Abstract

**Background:**

The high accuracy and precision of cerebral spinal fluid (CSF) biomarkers for the assessment of Alzheimer's Disease (AD) pathology has allowed regulatory approval and global clinical acceptance of these tools. The most advanced CSF assays include Aβ1‐42, Aβ1‐40 and its ratio, and phosphorylated Tau species including pTau181 and total Tau. To assess the interlaboratory accuracy of Aβ1‐42, Aβ1‐40 and its ratio on the Fujirebio Lumipulse system, 15 samples were tested by both Kentucky Clinical Trials Laboratory in Louisville, Kentucky and Teddy Clinical Research Laboratory in Shanghai China.

**Method:**

Clinical samples were selected to cover the dynamic range of the assays. As part of a phase 3 trial, fifteen (15) patient samples were collected, via lumbar puncture using the drip method. The patient sample was separated into multiple aliquots and one aliquot was sent to each laboratory. Each laboratory analyzed all aliquots same‐day, approximately six months apart, using Aβ1‐42 and Aβ1‐40 test kits—verified by passing two levels of quality controls (QC) on the day of analysis. The Aβ1‐42/Aβ1‐40 ratio values from each laboratory were statistically compared by Deming linear regression analysis. The Aβ1‐42/Aβ1‐40 ratio value interpretations were assessed for agreement.

**Result:**

The Deming linear regression analysis of CSF Aβ1‐42/Aβ1‐40 ratio values produced the following results, 95% confidence interval in parentheses, Slope 0.964 (0.890 to 1.037), Intercept 0.0011 (‐0.0049 to 0.0072), Std Err Est 0.0035 and Correlation Coefficient (R) 0.9918. Positive/abnormal (ratio ≤0.058)(*n* = 5), Likely positive (ratio 0.059 to 0.072)(*n* = 2) and Negative (ratio ≥ 0.073)(*n* = 8) interpretations indicate 100% agreement between the two laboratories.

**Conclusion:**

The CSF Aβ1‐42/Aβ1‐40 ratio results from two independent laboratories are statistically identical. A slope of 0.964, with 95% confidence interval including 1.00, and an intercept of 0.0011, with 95% confidence interval including 0.0, indicate a statistically significant positive correlation between the ratio values obtained in each laboratory. Additionally, Positive/abnormal, Likely Positive and Negative CSF Aβ1‐42/Aβ1‐40 ratio interpretations are identical between each laboratory.

